# Evaluating an addiction medicine unit in Sudbury, Ontario Canada: a mixed-methods study protocol

**DOI:** 10.1186/s12913-023-10313-0

**Published:** 2023-12-06

**Authors:** Kristen A. Morin, Karla Ghartey, Adele Bodson, Alexandra Sirois, Tara Leary

**Affiliations:** 1 Health Sciences North, Sudbury, ON Canada; 2 ICES North (Institute for Clinical and Evaluative Sciences), Sudbury, ON Canada; 3 Northern Ontario School of Medicine University, Sudbury, ON Canada; 4 University of Toronto, Toronto, ON Canada; 5 Cambrian College, Sudbury, ON Canada; 6 Laurentian University, Sudbury, ON Canada

**Keywords:** Mixed methods study, Addiction medicine, Program evaluation, Substance use disorders, Inpatient

## Abstract

**Background:**

In response to the escalating global prevalence of substance use and the specific challenges faced in Northern Ontario, Canada, an Addiction Medicine Unit (AMU) was established at Health Sciences North (HSN) in Sudbury. This protocol outlines the approach for a comprehensive evaluation of the AMU, with the aim of assessing its impact on patient outcomes, healthcare utilization, and staff perspectives.

**Methods:**

We conducted a parallel mixed-method study that encompassed the analysis of single-center-level administrative health data and primary data collection, including a longitudinal observational study (target n = 1,200), pre- and post-admission quantitative interviews (target n = 100), and qualitative interviews (target n = 25 patients and n = 15 staff). We implemented a participatory approach to this evaluation, collaborating with individuals who possess lived or living expertise in drug use, frontline staff, and decision-makers across the hospital. Data analysis methods encompass a range of statistical techniques, including logistic regression models, Cox proportional hazards models, Kaplan-Meier curves, Generalized Estimating Equations (GEE), and thematic qualitative analysis, ensuring a robust evaluation of patient outcomes and healthcare utilization.

**Discussion:**

This protocol serves as the foundation for a comprehensive assessment designed to provide insights into the AMU’s effectiveness in addressing substance use-related challenges, reducing healthcare disparities, and improving patient outcomes. All study procedures have been meticulously designed to align with the ethical principles outlined in the Tri-Council Policy Statement: Ethical Conduct for Research Involving Humans. The findings will be disseminated progressively through committees and working groups established for this research, and subsequently published in peer-reviewed journals. Anticipated outcomes include informing evidence-based healthcare decision-making and driving improvements in addiction treatment practices within healthcare settings.

## Background

In recent years, the global prevalence of substance use has reached alarming proportions, necessitating a comprehensive and evidence-based approach to address its multifaceted challenges [1]. Substance use poses a considerable public health concern in Northern Ontario, Canada, particularly in light of the restricted availability of healthcare service providers [2–5]. Data sourced from Public Health Ontario underscores the alarming disparity in opioid-related emergency department (ED) visits and fatalities within Public Health Sudbury & District, with figures consistently exceeding provincial averages [6]. Notably, Sudbury & Districts exhibited the highest per capita rates of opioid overdoses and related fatalities in Ontario during 2020, coupled with an elevated frequency of emergency department (ED) visits attributable to opioid overdoses and the toxic unregulated supply of drugs [6]. This trend has exhibited a persistent year-on-year escalation. To highlight, in 2019, the opioid overdose fatality rate stood at 28 per 100,000 individuals. However, by 2020, this figure surged to 52.4 per 100,000 individuals. This rise in statistics has exerted immense strain on acute healthcare services in the region, manifesting as a substantial influx of patients into the ED and a pronounced increase in hospital admissions [6].

Engaging individuals where they are at in their journey and encouraging addiction-related interventions in acute care settings, after medical stabilization has been linked to decreased emergency department utilization and more seamless transitions into outpatient treatment [7–10]. Existing literature suggests enhanced engagement with primary care and HIV treatment, decreased homelessness, and reduced drug use following discharge for individuals with who use drugs that receive addiction-specific consultations in hospital, as opposed to those who do not [11–14]. Despite the increasing incidence of substance use cases in hospitals and the advantages of interacting with patients during hospital visits, issues tied to substance use often remain unaddressed [15–18]. With absent or limited hospital-based support, patients are left to navigate their post-hospital care independently, potentially impeding the uptake of long-term treatment and contributing to the extensively documented high rates of hospital and ED readmissions [6, 19–21].

An Addiction Medicine Unit (AMU) has emerged as an essential component of in-patient care at Health Sciences North (HSN), an academic health science centre in Sudbury, Ontario, Canada. The AMU offers specialized addiction support that integrates medical and psychosocial interventions along with providing connections to community supports to effectively stabilize patients and manage addiction-related issues using a harm reduction philosophy. As the demand for the unit continues to rise, it becomes imperative to rigorously evaluate its efficacy and impact to ensure optimal patient outcomes and resource allocation.

This protocol lays the foundation for a comprehensive evaluation the AMU, aiming to provide valuable insights into its effectiveness, patient and staff satisfaction, and integration within the health care system. By adhering to rigorous research methodologies and considering a range of outcome measures, this evaluation will contribute to the ongoing improvement and optimization of addiction medicine units, ultimately leading to better outcomes for individuals grappling with substance use and related challenges. Specifically the objectives of the evaluation are as follows: (1) compares ED and hospital re-visits for patients receiving hospital-based substance-use support compared to those who received the standard of care at Health Sciences North in Sudbury, Ontario, Canada; (2) Evaluate the influence of the AMU on trends in health service utilization among patients admitted to the AMU through analysis of hospital administrative data; (3) Gain insight into patients’ viewpoints by conducting individual qualitative interviews to discern the perceived obstacles and essential elements of the AMU; (4) Gain insights into staff perspectives by facilitating focus groups to identify perceived obstacles and crucial elements of the AMU from the standpoint of the staff; (5) Evaluate changes in substance use, health risk behaviors, and social capital among AMU patients during their admission by conducting individual quantitative interviews both upon intake and discharge; (6) Evaluate changes in substance use, health risk behaviors, and social capital among AMU patients subsequent to their admission by conducting individual quantitative interviews one week, four weeks, and eight weeks after discharge from the AMU.

## Methods

### Design

This is an ongoing study mixed methods observation research design, centering on a cohort of patients who were admitted to the AMU at Health Sciences North (HSN) in Sudbury, Ontario, Canada. The evaluation period spans from March 10, 2021, to December 31, 2024.

### Setting

The AMU is a 20-bed medical unit located at HSN in Sudbury, Ontario. Established on March 10th, 2021, the unit operates under the guiding principles of HSN’s Harm Reduction philosophy and is dedicated to delivering specialized care for individuals at various stages of stability in their substance use. Comprehensively addressing both medical and psychosocial needs, the unit offers wrap-around care with a strong focus on medical and addiction treatment. The dedicated team comprises specialized professionals, including addiction medicine physicians, nurses, allied health practitioners, and peer engagement specialists, all collaboratively working towards improved patient outcomes.

Situated within the hospital, the unit allows patients from medical and psychiatric departments to receive ongoing medical care while concurrently benefiting from essential addiction support. Admission mandates a physician’s recommendation from a hospital unit, subsequently requiring acceptance by the AMU’s attending physician. The unit remains committed to providing addiction support irrespective of whether patients continue to use drugs or not. It is in place to prevent a person’s substance use from hindering their medical care and hospital stay. Furthermore, it plays a pivotal role in alleviating bed occupancy pressures on medical units and offers assistance to patients who encounter challenges accessing outpatient therapy due to their complex social circumstances. By ensuring patients complete their medical stay and extending addiction support until discharge, the unit fosters critical connections with community partners to facilitate continued care post-discharge.

To ensure appropriate patient placement within the confines of the AMU’s capabilities, multiple considerations must be factored in. These limitations encompass the lack of oxygen or suction capabilities, weight restrictions, and building code prerequisites that limit the admission of wheelchair-bound patients. The process of admitting a patient to the AMU commences with the referring unit identifying individuals with substance use concerns who continue to require hospital admission and would also benefit from the AMU’s services. The patient’s Most Responsible Physician (MRP) will liaise with the Addictions Physician to handover care. Should the MRP not endorse the decision to transfer, an Addiction Medicine Consult Service (AMCS) referral can be entered to provide assistance and engage in substance use-related care while the patient remains in their current unit. In the event the MRP approves the transfer, a physician to physician handover is completed, and an order entry is initiated. Subsequent to this, close collaboration occurs among the AMCS team, social worker, and addiction worker to assess the patient’s suitability according to admission criteria. Upon determining the patient’s suitability, the transfer can take place. The bed flow system is then notified about the acceptance of the patient for transfer to the AMU. A handover takes place between the physician and a nurse-to-nurse report is provided to support seamless care transitions. Finally, the patient is transferred to the AMU, where they can receive the specialized care, they require.

### Participants

The study includes all patients receiving treatment within the AMU at HSN. Referrals to the unit are initiated by the admitting physician, contingent upon the clinical criteria indicating a substance use disorder (SUD). Admission to the AMU necessitates an acute medical or psychiatric diagnosis, along with a concurrent need for ongoing care due to active addiction concerns or acute withdrawal requiring medical monitoring, outside the intensive care unit (ICU). We estimate that approximately 400 patients will be included with retrospective administrative data (objective 1), approximately 1,200 patients for the comparative effectiveness against the standard of care (objective 2) approximately 25 patients for the qualitative data collections (objective 3), 20 staff will be included (objective 4), 100 patients for the quantitative interviews (objective 5 and 6).

### Data sources, collection, and measurement

Timeline for objectives one through 6.

**Table:** 

Objective	Timeline
Protocol preparation	September 2022 to March 2023
Ethics application and approval	March 2023
Peer researcher onboarding	January 2024
Research staff training	January 2024 (and ongoing)
Objective 1 – ED and hospital re-visits in amu vs. standard care patients: cohort study using administrative data	June 2023
Objective 2 - AMU impact on health service utilization trends	September 2021 to August 2024
Objective 3 - Patient perspectives on amu: qualitative interviews	January 2023 and October 2023
Objective 4 - Staff perspectives of the AMU focus groups	June 2023 to September 2023
Objective 5 - Changes in AMU patients: pre- and post-admission quantitative interviews	April 2023 to October 2023
Objective 6 – Changes in AMU patients post discharge quantitative interviews	March 2024
Data curation and analysis	Quarterly September 2021 to August 2024
Final data analysis	October 2023
Manuscript writing and review	October to December 2023
Conference presentations	October 2023
Submitting for publication	September to December 2023

#### Objective 1

Compare emergency department (ED) and hospital re-visits for all patients receiving AMU compared to those who received the standard of care. This analysis is completed and was submitted for publication in June of 2023 [22]. We performed a retrospective observational analysis utilizing administrative data encompassing all patients who presented with an indication of substance use at Health Sciences North between January 1, 2018, and August 31, 2022. The primary endpoint was defined as an ED or hospital revisit within 30 days of the initial visit. The secondary endpoint encompassed all documented ED or hospital revisits observed within study period.

##### Study procedures and data collection

Participants were identified from HSN medical records spanning January 1, 2018, to August 31, 2022, and outcomes were tracked until September 30th, 2022. The Discharge Abstract Database (DAD) [15] provided hospital admission details, and the National Ambulatory Care Reporting System database (NACRS) [16] contains ED visit data including ICD-10 [17] diagnosis codes, serving as primary sources. Two standard-of-care groups were used as comparators: (1) The ED visit group - patients discharged or directly hospitalized from the ED without addiction support, and (2) the admit/no service group - inpatient admissions without specialized addiction care. Index events were defined as discharge dates with DAD/NACRS codes F10-19 within International Classification of Disease (ICD)-10-CA Chap. 5 [13]. Study outcomes, set a priori, were ED or hospital visits within 30 days of an index event [8]. The 30-day window started with index visit discharge; if readmission was absent, the window restarted with the next admission.

##### Data analysis

Descriptive analyses summarized baseline characteristics using mean, standard deviation, frequencies, and percentages for continuous and categorical variables [18]. Logistic regression models were used to assess associations between main interventions, covariates, and outcomes, accounting for within-patient variability with random effects in 30-day windows [19]. Cox proportional hazards models [20, 21] investigated associations of main interventions and covariates with time to readmission within 30 days and time to first revisit, incorporating random effects for within-patient variance for the former. Kaplan-Meier curves were employed to show raw revisit probabilities within 30 days of index admission and time to first revisit within the first-year post-index admission. These curves were tested for differences using the Mantel-Haenszel test. Statistical analyses were performed using R version 4.2.2 [22].

#### Objective 2

Evaluate the influence of the AMU on trends in health service utilization among patients admitted to the AMU through analysis of hospital administrative data for all patients admitted to the AMU. We are performing an ongoing observational analysis utilizing routinely collected data at HSN, spanning from March 1, 2020 (one year prior to the AMU’s implementation) to December 31, 2023. Data is gathered through the hospital’s electronic medical record (EMR) system. In this analysis, all patients affiliated with the AMU at HSN will be included, thereby obviating the need for recruitment or consent due to the inherent nature of the data.

##### Study procedures and data collection

We put forth a simulated dataset comprising variables derived from routinely collected data at HSN. Subsequently, collaborated with the Decision Support personnel to extract the data in a format conducive to generating quality improvement reports and facilitating trend analysis. These reports continue to be generated by the Decision Support team using the Business Intelligence Tool, accessible exclusively to authorized HSN personnel. On a quarterly basis, the reports have been subject to analysis utilizing the SAS for Academics version. The resultant aggregated statistics continue to be presented to the management and staff of the AMU, as well as the executive team at HSN. A dashboard will be created to depict trends in the data over the course of the collection period.

##### Data analysis

After data collection is complete (December 31, 2023), the research team will undertake an interrupted time series analysis concerning health service utilization and referral patterns over time. The primary objective of this analysis is to assess the extent to which the implementation of the AMU has influenced acute health service utilization at Health Sciences North in Sudbury, Ontario. Descriptive metrics will be employed to delineate patient characteristics. Subsequently, regression models will be applied to assess the associations between patient factors, program variables, and the frequency of health service utilization.

#### Objective 3

Gain insight into patients’ viewpoints by conducting individual qualitative interviews to discern the perceived obstacles and essential elements of the AMU. We conducted qualitative interviews with patients to gain insight into their perspectives regarding the unit’s key components, their satisfaction levels, and the perceived benefits derived from the unit. Assessing patient satisfaction holds significant value in informing healthcare priority decisions. All patients who provide consent by signing the informed consent form will be included in the analysis.

##### Study procedures and data collection

During their AMU stay, patients had the chance to participate in the study, presented by AMU or research staff. Research staff were available weekly, to recruit patients. Interested patients could proceed with immediate interviews or schedule later sessions. Research staff obtained patient signed consent, then conducted interviews either via Microsoft Teams or in person, utilizing key questions. Compensation included $10 gift card for an onsite coffee shop. Interviews were audio-recorded, transcribed, and stored securely. Data collection continued until saturation or 50 interviews. Practical considerations, such as the availability of participants within the specified timeframe and the resources required for data collection and analysis, were taken into account. Thematic analysis identified patterns, shared with AMU and HSN for quality enhancement, and for scientific publication.

#### Objective 4

Gain insights into staff perspectives by facilitating focus groups to identify perceived obstacles and crucial elements of the AMU from the standpoint of the staff. We will evaluate staff perspectives regarding the program’s key components and perceived benefits of the AMU. All AMU staff will have the opportunity to participate in the focus groups.

##### Study procedures

Focus groups were organized involving AMU staff members. Participation was voluntary, and interested participants signed a consent form to allow data usage. Upon survey completion, participants received a $10 gift card to a local coffee shop. The research team, in collaboration with management, developed the focus group questions and schedule to ensure all staff members were provided an opportunity to join. The focus group sessions were recorded with a recorder and then uploaded to Microsoft Teams for transcription. A research assistant verified the electronic transcription by listening and ensuring data accuracy. Focus groups were audio-recorded, transcribed, and stored securely. Thematic analysis identified patterns shared with AMU and HSN for quality improvement purposes.

#### Objective 5

Evaluate changes in substance use, health risk behaviors, and social capital among AMU patients during their admission by conducting individual quantitative interviews both upon intake and discharge.

Our objective is to perform pre/post (baseline and discharge) data collection in order to evaluate alterations in outcomes of interest during patients’ AMU stays. This will be accomplished through chart extractions and one-on-one patient interviews.

##### Recruitment and consent

Patients are presented with the opportunity to participate shortly after their admission to the unit. Every patient in the unit is given the chance to join the study. Staff provides a brief overview of the study using the study script, and patients can choose to engage in the interview immediately or schedule it for a later time. Before conducting the interview, research staff guide patients through the informed consent process. Patients are asked to provide signed consent for two aspects of the study: (1) to use data extracted from their intake form as a baseline measurement, and (2) to participate in an interview either at the present moment or upon discharge from the unit, with the information provided accordingly. Participants receive a copy of the consent form.

##### Data collection

The one-on-one interviews are conducted in person by a peer support worker. Patients receive a $10 gift card to an onsite coffee shop as compensation. Upon intake, the staff conducts a mental health and addiction history which includes components of the Maudsley Addiction Profile, a tool developed for outcomes research with persons with problematic substance use. The second aspect of the baseline measure involves the utilization of the Recovery Capital Index (RCI) [22]. During the discharge phase, the RCI measure will again be administered, alongside the post-data collection forms. A data set and data dictionary were created to ensure standard data entry from charts and other data collection forms. Research staff input the information into a password-protected datasheet omitting patient names. A list of patient names and IDs is securely stored in a separate locked cabinet. Data collection will continue until 100 patients are recruited. Research findings will be aggregated and presented to AMU management, staff, and the executive team at HSN for quality improvement initiatives. Additionally, the data will be used for scientific publication.

##### Sample size justification

The sample size calculation was based on several key considerations: the primary outcome measures, the desired level of confidence, the margin of error, and the effect size.

The primary outcome measures the implementation of strategies to reduce substance use risk among individuals admitted to the AMU. A confidence level of 95% (α = 0.05) was chosen. There is limited research looking at inpatient addiction medicine units, so we conservatively estimated a medium effect size to ensure that the study is adequately powered to detect meaningful differences. A power analysis was conducted to determine the probability of detecting a statistically significant effect, assuming the chosen sample size. With a sample size of 74 participants, the study achieves a power of 80% or higher to detect the expected effect size at the 5% significance level. Practical considerations, such as the availability of participants within the specified timeframe and the resources required for data collection and analysis, were also considered.

##### Data analysis

After recruitment is complete, descriptive metrics will be employed to delineate patient characteristics. Subsequently, Generalized Estimating Equations (GEE) will be used to compare and analyze the repeated measures (intake/discharge).

##### Objective 6

Evaluate changes in substance use, health risk behaviors, and social capital among AMU patients subsequent to their admission by conducting individual quantitative interviews one week, four weeks, and eight weeks after discharge from the AMU. This will be a mixed-methods study in the form of a quantitative prospective longitudinal study and qualitative interviews.

##### Recruitment and consent

A core principle of this phase of the evaluation is to actively engage people who use drugs (PWUD) in order to meaningfully prioritize the needs of the research participants. Patient recruitment will begin during their AMU unit admission and will be confirmed upon their discharge. After discharge, we will collect contact information to facilitate scheduling meetings in person, over the phone, or virtually. Community agencies receiving referrals from the AMU will also be engaged to assist with post-discharge participant contact (with participant consent). Honorariums reflecting a living wage will be provided at every post-discharge contact. This financial compensation is particularly important when working with PWUD, who statistically experience higher poverty rates and lower education levels as outlined by the Canadian Association for People Who Use Drugs (CAPUD) [23, 24].

##### Data collection

Semi structured interviews will be conducted one week, four weeks, and eight weeks after discharge from the AMU. Qualitative questions will be constructed in collaboration with PWUDs. The Maudsley Addiction Profile [25] and the Recovery Capital Index (RCI) [26] will be administered during the interviews. A list of patient names and IDs will be securely stored in a separate locked cabinet. Data collection will continue until 100 patients are recruited. We’ve established a priori an allowance of 2% lost to follow up and in that case we will simply omit those cases with the missing data and analyze the remaining data. This approach is listwise deletion. In the case that over 2% of cases have been lost to follow up, we will use imputation techniques to preserve all cases by replacing the missing data with a probable value estimated by other available information. After all missing values have been replaced by this approach, the data set is analyzed using the standard techniques for complete data. Research findings will be aggregated and presented to AMU management, staff, and the executive team at HSN for quality improvement initiatives. Additionally, the data will be used for scientific publication.

### Statistical analysis

Quantitative Data: After recruitment is complete, descriptive metrics will be employed to delineate patient characteristics. Subsequently, Generalized Estimating Equations (GEE) will be used to compare and analyze the repeated measures (intake/discharge).

Qualitative Data: as stated previously, the importance of PWUDs being involved in the project is vital. Along with researchers, PWUDs will be involved with the qualitative analysis using a grounded theory approach, as it enhances the research process. Their knowledge and perspectives of inequitable barriers and navigation through the system as an oppressed group is unique from having lived or living through something, that a person who has not, can never have [27].

### Research staff training

The study staff underwent comprehensive training to ensure full competence in all assessments and procedures. The mandatory training encompassed the Tri-Council Policy Statement: Ethical Conduct for Research Involving Humans (TCPS-2), in addition to protocol-specific training as required, such as assessments, data management, and collection. New staff members participated in a General Orientation session provided by HSN. This orientation covered general policies, procedures, and operations that applied. They also became familiar with HSN Research Institute policies and acknowledged their commitment to adhere to them. Before engaging in recruitment, screening, and participant enrollment, all study staff had to fulfill the training prerequisites. Training was an ongoing process, spanning various stages of the study, including pre-study preparations and implementation. Interactive face-to-face sessions and self-study were employed to deliver all tool-related training.

### Data governance, peer review and ethical considerations

We will respect the Tri-Council Policy Statement, Chap. 1, which highlights the importance of respect for persons, concern for welfare, and justice throughout all phases of the research process. Anonymized data collected at HSN will be linked in accordance with data governance protocols.

Peer review was undertaken in both the funding and ethics approval for this project. Funding was reviewed and approved by the Northern Ontario Academic Medicine Association review committee; ethics application and protocol were reviewed by the Health Sciences North Research and Ethics Board.

### Co-design, knowledge integration and translation

Co-design stands as a fundamental principle within this evaluation. An embedded researcher at HSN took the lead in collaborating directly with leadership to co-create the evaluation framework and research designs. The involvement of staff and peer workers is pivotal, as they contributed to the development of data collection tools, with peers leading the data collection efforts for this project. Regular input from the leadership team, frontline staff, and peer support workers, who possess practice-based knowledge, will ensure the contextualization of research findings, will facilitate the successful implementation of these findings, and provides timely feedback on the process.

The knowledge translation of research findings will be centered on disseminating the program’s activities to stakeholders and making research findings accessible to the public. HSN’s research and communications team will devise knowledge translation strategies aimed at sharing research outcomes with the public. These strategies encompass leveraging social media platforms to distribute infographics and concise one-page research summaries. Additionally, an interactive dashboard will be developed to provide live updates on ongoing health service utilization trends.

## Discussion

The protocol outlines a comprehensive framework for the evaluation of the AMU within a healthcare setting in Northern Ontario, Canada. The AMU addresses the pressing challenges posed by PWUD, offering specialized care that bridges the gap between medical treatment and addiction support. By conducting a mixed-methods approach, this evaluation aims to provide a holistic understanding of the AMU’s impact on patients’ health outcomes, healthcare utilization, and overall well-being.

The protocol emphasizes the significance of patient-centered perspectives by including qualitative interviews, ensuring that the voices and experiences of those utilizing the AMU services are integral to the evaluation process. Simultaneously, quantitative analyses will provide a robust assessment of the AMU’s effects on healthcare utilization trends, contributing valuable insights for evidence-based healthcare decision-making.

Furthermore, the study recognizes the importance of staff perspectives, offering a comprehensive view of the AMU’s functioning, effectiveness, and potential areas for improvement. By engaging both patients and staff, this evaluation aims to yield actionable insights that enhance the AMU’s role as a crucial component of addiction treatment and stability.

This evaluation seeks to shed light on the role of the AMU in addressing issues related to substance use, reducing healthcare disparities, and improving patient outcomes. The insights garnered from this study will have implications not only for the specific healthcare institution but also for addiction medicine practices across broader contexts. Ultimately, this protocol sets the stage for a comprehensive and valuable evaluation that can drive positive change in addiction treatment and healthcare delivery.

## Data Availability

Data sharing is not applicable to this article as no datasets were generated or analyzed during the current study.

## Abbreviations

AMCS: Addiction Medicine Consult Service
AMU: Addiction Medicine Unit
CAPUD: Canadian Association for People Who Use Drugs
DAD: Discharge Abstract Database
ED: Emergency department
EMR: electronic medical record
GEE: Generalized Estimating Equations
HSN: Health Sciences North
ICD: International Classification of Disease
ICU: intensive care unit
MRP: Most Responsible Physician
NACRS: National Ambulatory Care Reporting System database
PWUD: People who use drugs
RCI: Recovery Capital Index
SUD: Substance use disorder
TCPS-2: Tri-Council Policy Statement:Ethical Conduct for Research Involving Humans
